# Supplementary material for: Total infectome characterization of respiratory infections in pre-COVID-19 Wuhan, China
Source: PLoS Pathog. 2022 Feb 17;18(2):e1010259. doi: 10.1371/journal.ppat.1010259 (PMC8853501; doi:10.1371/journal.ppat.1010259)
Supplement: Attachment — Submitted filename: Response.docx [file ppat.1010259.s003.docx]

**Re: Shi *et al*. PPATHOGENS-D-21-01755 ‏ “Total Infectomes Characterization of Respiratory Infections in pre-COVID-19 Wuhan, China”**

Dear Editor,

We thank you and the reviewers for the comments and suggestions on this paper. We have addressed and incorporated all the reviewers’ requests and recommendations into a new revision of the paper that is enclosed here. We have also provided a point-by-point response to all the comments made. Finally, we have uploaded an MS Word ‘Compare’ document that shows all the changes made.

We hope that you will now consider our revision of appropriate quality to be published in *PLoS Pathogens*.

Once again, we thank you for your consideration.

Yours sincerely,

Yong-Zhen Zhang

PhD and adjunct Professor

School of Life Sciences

Fudan University, Shanghai, China

[zhangyongzhen@fudan.edu.cn](mailto:zhangyongzhen@fudan.edu.cn)

Tel: 086-21-57249022

Fax: 086-21-57248782

**Referees' comments:**

**Referee #1:**

The manuscript by Shi et al presents the viruses, bacteria, and fungi that can be found in acute respiratory infections / pneumonia patients living in Wuhan area in the years 2016 and 2017. The NGS data on the infecting pathogens is presented, the “infectome”. Several pathogens were found, yet no SARS-CoV-like viruses. The absence of SARS-CoVs confirms earlier reports that PCR-checked samples collected in 2019 in Wuhan (Kong et al . <https://www.nature.com/articles/s41564-020-0713-1>). The manuscript provides a clear presentation of the pathogens that did play a prominent or a less prominent role in the respiratory infections. The study is clear and the paper is well written.

**Response:** We thank the reviewer for their constructive comments. Our responses are given in the blue text, and changes/additions to the manuscript are shown in blue italics.

Some parts may need some extra text or explanations:

1. - Page 6, line 100-101. Please add how RPM is defined.

**Response:** The definition is now provided between lines 106-107, to read:

*“…**The abundance threshold for pathogen positives was set as* *1 sequence read per million (or RPM).…”*

2. - Page 6, line 100-101. It is unclear why a cut off of 1000 RPM or 1 RPM is taken for unknown and known viruses respectively. Some clarification is needed.

**Response:** No new pathogens were discovered at either 1000 RPM or 1 PRM suggesting that these thresholds are appropriate. The corresponding text has been revised between lines 105-108, to read:

*“**…**For the latter, we only considered DNA and RNA viruses that are related to a virus genus or family that have previously been shown to infect mammals.* *The abundance threshold for pathogen positives was set at 1 sequence read per million (or RPM). In addition, likely commensal bacteria were not considered here….”*

3. - Page 6 line 114-117. Most probably the *E.coli* reads in figure 2, in cases and controls, are contaminations or reads originating from library-prep ingredients. Presumably the reads were also found in blank controls, and the manuscript would improve if presence of *E.coli* in all samples (and blanks?) is mentioned, and explanation is added.

**Response:** We agree that the *E. coli* reads likely originate from contamination because they appear in blank controls. The relevant text has been added to lines 120-123, to read:

*“Finally, none of the pathogens described here appeared in the blank sequencing controls. An exception was Escherichia coli, which appeared in the experimental, healthy control and blank control groups (1296-2173 RPM), but was regarded as likely contamination and so not considered further.”*

4. - Page 14, lin 284: samples from healthy volunteers: Please include in which month of which year the control healthy persons were sampled. Was this also in 2016-2017? If sampled in a different year, then this could be mentioned in the discussion as a weakness in the study design

**Response:** Samples from healthy controls were taken between March 27^th^ to June 15^th^ 2017, which overlaps with the time when samples from the experimental groups were taken. The corresponding text has been added to lines 288-290, to read:

*“**To establish a healthy control group, 5ml-10ml of BALF samples were also taken from 32 volunteers without respiratory symptoms between March 27^th^ to June 15^th^, 2017.”*

5. - Page 15, line 303-304. RPM: it is not clear what the reads per million exactly represents (per million of what?). Since cut-offs are based on RPMs this needs clear explanation

**Response:** We apologize for the lack of explanation. RPM stands for Reads Per Million of the total number of reads in the library. This information has been added to lines 309-311, to read:

*“**Viral abundance was summarized from both analyses, calculated using the relation: total viral reads/total non-redundant reads* 1 million (i.e.,* *r**eads per million of total non-redundant reads, RPM)”*

6. - Page 15 line 321. Table SX, is probably Table S1

**Response:** Corrected.

**Referee #2:**

The manuscript by Shi *et al* is a traditional viral metagenomics study looking at BALF samples from 408 respiratory cases and 37 healthy controls collected 2016-17 from Wuhan. It is expertly done including analyzing total RNA including those of viruses, bacteria, and fungi. Mostly traditional human viral pathogens are identified as well as bacterial and fungal pathogens. Of note a zoonotic infection with an avian bacterial pathogen was detected as well as enterovirus D68. The wide range of viruses found is interpreted as reflecting Wuhan's connectivity with rest of China and the world.

Major issues:

1. Technically this study is well performed and issues such as contamination and index hopping are appropriately addressed and the conclusions well supported.

31 No SARS CoV2 RNA was detected but because NGS is generally less sensitive than a good RT-PCR the authors should discuss whether al the RNAs were also analyzed by qPCR. It is conceivable that a still poorly replicating SARS2 precursor was replicating in human but a low viral loads not detectable by NGS. It seems it would have been easy to add that qPCR to the long list of qPCR already used used.

**Response:** We agree with the reviewer that SARS-CoV-2 screening is important for confirming that this virus is not present in the sample. We therefore performed SARS-CoV-2 PCR testing on all the samples. This information has been added to lines 329-331 in the Methods section, to read:

*“**In addition, the qRT-PCR assays with primers targeting the ORF1ab and N genes of SARS-CoV-2 were performed on these samples using* *a commercial COVID-19 nucleic acid detection kit (DaAn Gene, China) (Cat. DA0932)”*

None of the samples were tested positive. This result has been added to lines 166-167, to read:

*“**Finally, the qPCR assays for* *SARS-CoV-2 provided negative results for all the samples examined here.”*

Minor issues:

2. Line 27 Would be interesting to hear whether or not such BALF were collected later than 2017.

**Response:** We agree that it will be interesting to examine the samples collected between 2018-2019, just before the COVID-19 pandemic. The BALF samples between 2018-2019 were indeed collected, including the samples from children hospital in Wuhan. Unfortunately, we have not finished the pathogen screening due to the current COVID-19 pandemic. In addition, the pathogen screening of the BALF samples between 2016-2017 had been finished prior to the SARS-CoV-2 emergence.

3. Introduction: line 72 Surely there are several prior human metagenomics studies of respiratory diseases that have been published and could be referred and compared to.

https://pubmed.ncbi.nlm.nih.gov/32497137/

https://pubmed.ncbi.nlm.nih.gov/33367583/

https://pubmed.ncbi.nlm.nih.gov/32872469/

etc

**Response:** We have now included these references as suggested. The corresponding text has been revised in lines 73-75, to read:

*“**The recent application of mNGS approaches have revealed a diverse range of pathogens and outlined the infectomes potentially associated with severe pneumonia^9^, lower respiratory tract infection^10^, and other respiratory diseases^8,11^.”*

4. Line 147 BLAF typo

**Response:** Corrected.

5. Line 153 Strikingly, strong correlations…. Plenty of prior publications have reported on this correlation. Include a few references here or in discussion.

**Response:** We agree and the corresponding description has been removed.

6. Line 232 change 106 to 106

**Response:** Unfortunately, we do not totally understand this point.

7. Clarify whether the confirmatory PCR were done on only the samples found positive by NGS or on all cDNAs? If so were any infections missed by NGS but seen by qPCR?

**Response:** The qPCR assays were performed on only positive samples because there was insufficient RNA for a comprehensive overall testing. The corresponding text has been revised between lines 327-329, to read:

*“**For RNA viral pathogen positive samples, the same sample that was positive for a specific pathogen was also subjected to a qRT-PCR assay with primers sets designed for this or related group of pathogens (Table S2)”*

**Referee #3:**

The scientific goal of this research (and thus the value of the findings) is not clear. There is no reason to suspect that SARS-CoV-2 could be circulating in humans before 2019. Thus, the value of a finding that it was absent in Wuhan in 2016 is not clear. The findings regarding infectome in pneumonia patients are not referred to infectomes elsewhere in the world or to any reference groups. There is no hypothesis how the infectome in 2016 could be linked to the SARS-CoV-2 emergence or the outbreak spread. A description of infectome in pneumonia patients may be interesting by itself, but has limited scientific impact and is more suitable for journals that do not consider scientific impact.

**Response:** We must respectfully disagree with the reviewer on this point. As we very clearly state in the paper, our aim was to document the respiratory infectome in patients with acute respiratory disease and pneumonia – a case definition very much like COVID-19 – in the one of the hospitals where COVID-19 was first described and treated. We also then make a number of explicit and general conclusions about the nature of respiratory infectomes. Hence, we firmly believe that our paper is of clear scientific importance outside of the context of COVID-19.

The phylogenetic analysis is ridiculous. While the methodology of tree reconstruction is acceptable, it was not detailed how a few dozen sequences out of thousands available in Genbank were chosen for the analysis. Without a clear and well explained reference sequence selection algorithm, it is not possible to judge the validity of conclusions.

**Response:** We apologize for not providing the rationale of choosing the reference sequences. We began with all reference sequences downloaded from GenBank that were associated with a specific taxonomic group (species or family). These were then placed into several clusters based on genetic similarities. We then selected those representative of phylogenetic diversity by randomly choosing one sequence from each of the clusters, and combined these with those that exhibited a close relationship with the viruses identified in this study. The details of reference sequences selection are now added to lines 337-342, to read:

*“**To choose corresponding reference sequences for phylogenetic analyses, all viral genomic data were first downloaded from NCBI/GenBank under a specific taxonomic group (species or family) and clustered based on genetic similarities (99% ~ 99.9% based on virus species). We then selected those sequences that were (i) representative of background genetic diversity from each of the clusters and (ii) the closest hits in the blast analyses to establish a reference data set.”*

In addition, each of the phylogenetic tree presented here is in reality a **subtree** from a much larger phylogeny. This was done so that the relevant details (strain names, geographic information, isolation year, amongst others) of the sequences most closely related to the viruses discovered here can be easily visualized.

Finally, we have updated the reference sequences so that they are current. The revised trees can be found in Figure 3.

**Referee #4:**

In this study, Shi *et al.* examined pneumonia and acute respiratory infections at the Central Hospital of Wuhan between 2016 and 2017 by sequencing the bronchoalveolar lavage fluid samples from 408 patients. They identified 37 pathogen species, comprising RNA viruses, DNA viruses, bacteria and fungi, defining a stable core infectome. The prevalence of an atypical respiratory virus EV-D68, and a single case of a sporadic zoonotic pathogen – Chlamydia psittaci was reported. These data also suggest that SARS-CoV-2 or closely related viruses were absent from Wuhan in 2016-2017. It is an important study in the context of investigating early events of COVID-19 transmission in Wuhan. But the analysis and presentation need further improvement.

Major issues:

1. Line 164 and Figure 4: What is the rationale of choosing the viral sequences to be presented on the phylogeny tree? Were they well-characterized or circulating in the world previously or during the time of sampling (2016 to 2017)? Since the viral sequences themselves can determine the tree, if there are not enough sequences from different geometric regions, the trend of HCoVs clustering with US sequences is not conclusive. It can simply be due to more US sequences in the database. A similar trend can be found in IVA and HPIV1 in Figure 4.

**Response:** Please see our response to Reviewer #3 above. Again, we began with all reference sequences downloaded from GenBank that were associated with a specific taxonomic group (species or family). These were then placed into several clusters based on genetic similarities. We then selected those representatives of diversity by randomly choosing one sequence from each of the clusters and combined these with those that exhibited a close relationship with the viruses identified in this study. In addition, each of the phylogenetic tree presented here is just a **subtree** from a much larger phylogeny. This was done so that the relevant details (strain names, geographic information, isolation year, amongst others) of the sequences most closely related to the viruses discovered here can be easily visualized.

We also agree with the reviewer that the trend of our HCoVs clustering with US sequences might be an artifact due to lack of reference sequences from other geographic locations. We have therefore deleted the text on this point.

2. The infectome analysis was not thorough enough. For example, 1) no genome coverage and depth was shown for any pathogen NGS results which can be more storytelling than RPM value alone. 2) Any correlation of age, sex, severity and pre-existing condition to the time of sampling or between each other? Any correlation of pathogens identified with age/sex/severity/pre-existing conditions?

**Response:** Genome coverage and depth are convertible. Furthermore, for DNA viruses, bacteria and fungi, since we are sequencing transcriptome instead of genome it is more appropriate to use RPM than genome coverage as a measure of abundance. The results of correlation analyses have been performed and provided in Table S1. Corresponding descriptions have been added to line 97-99 and lines 126-127, to read:

*“**No correlation was found between age, gender, severity, and sampling time. One exception was age and gender, with the male patients* *(range 16-90, medium 63) were slightly older than female patients (range 16-85, medium 59) (Table S1).”*

*“Notably, none of age, gender, sampling time and disease severity of patients showed significant effect on the abundance of all identified pathogens (Table S1).”*

3. The key focus was understantably on SARS-CoV-2. But did the authors also examined the presence of other SARS-related CoVs? It will be useful to state/discuss this.

**Response:** Since we used a read blast (both blastn and Diamond blastx) method to identify potential pathogens, we examined every single read for their potential close or distant relationship with SARS-CoV-2 and SARS-like viruses. The corresponding discussion can be found in lines 194-200, to read:

*“**Our unbiased metagenomic survey in patients presenting with pneumonia or acute respiratory infection provides strong evidence that SARS-CoV-2 or any related SARS-like viruses were absent in Wuhan approximately two years prior to the onset of pandemic, although a variety of common cold coronaviruses (HKU1, OC43, 229E, and NL63) were commonly detected in our cohort.* *Indeed, the earliest COVID-19 case, identified by qRT-PCR or next-generation sequencing-based assays performed at designated authoritative laboratories, can currently only be traced back to December 2019 in Wuhan^12^.”*

Minor issues:

1. Line 94: For each patient, was the BALF sample taken upon hospitalization or some time afterwards? Clarify this as it would be important for the timing of NGS.

**Response:** We have added the information between lines 96-97, to read:

*“**The average time between hospitalization and sample collection was 3.36 days (range 0-33, medium 3)”*

2. Line 107: Please clarify co-infection. Should it be two different pathogens (for example, two different RNA viruses) or two different category pathogens (for example, one virus and one bacteria)?

**Response:** It should be two different species of pathogens regardless of virus, bacteria or fungi. The relevant text has been revised in lines 113-114, to read:

*“**Co-infection with two different pathogen species was also commonplace, …”*

3. Line 124 and Figure 2C: For each category (RNA/DNA Viruses, Bacteria and Fungi), what is the order for each pathogen? Some are ranked alphabetically (Bacteria), which makes no sense. Should be ordered according to the taxonomy (Order or Family) or prevalence.

**Response:** We thank the reviewer for pointing this out. The pathogens are now reordered based on taxonomy, and the corresponding figure (i.e., Figure 2) has been revised.

4. Line 151: “For each of the pathogens identified here”, please clarify “here”. Does it mean all 37 pathogens were validated through qPCR, and were all these results shown?

**Response:** We apologise for the confusion here: we only confirmed the presence of RNA viruses, and only pathogen positive samples were used for the qRT-PCR assays (otherwise there would not be sufficient RNA). The relevant text has been revised to reflect the scope and target of the confirmation work performed. Please see lines 327-329, to read:

*“For RNA viral pathogen positive samples, the same sample that was positive for a specific pathogen was also subjected to a qRT-PCR assay with primers sets designed for this or related group of pathogens (Table S2)”*

5. Figure 1C: The Y-axis should labeled “Age” and not “No of cases”.

**Response:** Corrected as suggested.

6. Figure 2A: Actual number and fraction not shown. Please show this information as a pie chart alone can be visually misleading.

**Response:** Corrected as suggested.

7. Figure 2B: Please show the actual number of cases for each bar.

**Response:** Corrected as suggested.

8. Figure 3: Please revise the x-axis range for IVA and HPIV3. A Ct value of less than 10 and above 40 is impractical. There are no qPCR results shown for bacteria and fungi, but Table S1 indicated so. Should show at least one for each category.

**Response:** Corrected as suggested. qPCR was only performed for RNA virus because there was insufficient RNA left for all the confirmatory work. Table S2 (original Table S1) has been revised and all bacterial primers were removed from the table.

9. Figure 5: The figure legend indicated only non-zero abundance levels are reported, but zero was actually shown on the figure for control groups (also according to the heatmap in Figure 2C). Is this non-zero abundance is only applied to the patient group?

**Response:** We apologize for these inconsistencies. The figure has been revised and the 0 data points in the control groups have been removed.

10. Line 281: 409 or 408 samples?

**Response:** We apologize for the mistake - it should be 408 samples. The text has been corrected.

11. Line 296-298: Version of the software and database not shown. What is the software used for the mapping?

**Response:** The version number has been added to software. The software used for mapping was Bowtie2. This information has been added to the Methods section in line 317*.*
